# Qualitative Differences and Emission Persistence of Volatile Organic Compounds from Bio-Based Particleboards

**DOI:** 10.3390/ma15155278

**Published:** 2022-07-30

**Authors:** Ramunas Tupciauskas, Kristine Meile, Daniela Godina, Janis Rizhikovs, Michail Syrpas, Petras Rimantas Venskutonis

**Affiliations:** 1 Latvian State Institute of Wood Chemistry, Dzerbenes 27, 1006 Riga, Latvia; kristine.meile@kki.lv (K.M.); daniela.godina@kki.lv (D.G.); janis.rizikovs@kki.lv (J.R.); 2 Department of Food Science and Technology, Kaunas University of Technology, Radvilenu pl. 19, LT-50254 Kaunas, Lithuania; michail.syrpas@ktu.lt (M.S.); rimas.venskutonis@ktu.lt (P.R.V.)

**Keywords:** lignocellulosics, steam explosion pretreatment, binder-less particleboard, suberinic acids-bonded particleboard, headspace solid-phase microextraction (HS-SPME), gas chromatography–mass spectrometry (GC–MS), volatile organic compounds (VOCs)

## Abstract

An attempt to reduce, replace, or even eliminate the synthetic resins from wood-based panels alongside broadening the array of raw lignocellulosics is still essential and attractive. Many pretreatments of lignocellulosics have been studied, among which steam explosion (SE) resulted in superior physical-mechanical properties of the obtained binder-less boards. However, the SE pretreatment leads to a relatively strong odor, which is even emitted from the obtained binder-less boards independent of the raw lignocellulosic, raising concern about the use of the boards. Emissions of volatile organic compounds (VOCs) were investigated in the framework of the study from binder-less boards obtained from different SE raw lignocellulosics and SE-untreated suberinic acids-bonded particleboard. VOCs were collected by headspace solid-phase microextraction (HS-SPME) and analyzed by gas chromatography–mass spectrometry (GC–MS) for 28 days with an interval of 2 weeks. The results showed that the number of detected VOCs and their chromatographic peak area varied significantly depending on the raw lignocellulosic, board density, and post-treatment (overlayering), decreasing over time. The lowest area of detected VOCs was demonstrated by the suberinic acids-bonded particleboard, while the highest area was detected from the high-density binder-less board obtained from SE hemp shives with the main compound of furfural (up to 70%) in all board types.

## 1. Introduction

Wood-based panels (WBPs) are important building construction materials widely used for many applications and are comprised of plywood, particleboards, fiberboards, and oriented strand boards, with the most produced and consumed capacity of particleboards (~50% in Europe) [1]. Commercial WBPs are produced from mechanically treated wood bonded by a synthetic adhesive usually containing formaldehyde that is continuously emitted during the exploitation, thus polluting the indoor air quality. A relatively new issue is the profile of volatile organic compounds (VOCs) that are also emitted from WBPs, raising no lower concern than formaldehyde as they contain terpenes and aldehydes, e.g., furfural, and organic acids characterized as indoor air pollutants [2]. As formaldehyde was recognized as a human carcinogen [3], the WBP industry was challenged to strictly regulate its level, limiting legislation in all developed countries. No mandatory upper levels are established yet for VOCs from WBP. However, Germany and France were pioneers in their regulation, contributing to the Construction Products Directive [4] and publishing specific regulations followed by Belgium [5].

Another important challenge of the WBP industry is the continuously changing raw material situation due to the global changes in the forest area, high competition, and growing demand for WBP production [1]. Therefore, agricultural lignocellulosics as nonfood by-products are very attractive from an economic and sustainability perspective for developing particleboards [6], particularly as binder-less boards [7,8,9]. This type of board consists of only a selected lignocellulosic that is mechanically processed by shredding and then hot-pressed under certain conditions at which lignin starts to melt and interact with other constituents, resulting in a binder-less composite. To improve the interaction between the constituents of lignocellulosics, many pretreatments have been studied; steam explosion (SE) resulted in superior physical-mechanical properties of the obtained binder-less boards [10]. However, the SE pretreatment leads to a relatively strong odor even emitted from the obtained binder-less boards independent of raw lignocellulosic species, which raises concern about the use of the boards. From this point of view, raw lignocellulosic species might play a key role as it was demonstrated that spruce strands showed the highest amounts of total VOCs while untreated hemp shive showed the lowest [11].

As a reaction to the industry’s challenge related to formaldehyde emission, bio-based adhesives are on the research top, generally comprising lignin, tannins, protein, and starch [12]. A very new and promising bio-based adhesive has been revealed in the form of suberinic acids containing binder from birch wood outer bark [13] investigated for application in particleboard [14] and plywood [15]. However, very few studies have investigated the VOC profiles of binder-less and bio-adhesive-bonded particleboards. It has been reported that pyrolysis bio-oil used to substitute phenol in phenol-formaldehyde resins for producing plywood partially presents greatly increased carcinogenic and noncarcinogenic risks because of the detected significantly higher emissions of formaldehyde, N,N-dimethylformamide, benzofuran, and furfural [16]. Chemical modification of raw hemp shives with tartaric acid, citric acid, and sodium bicarbonate resulted in an adverse effect, leading to increased total VOC emissions [11]. Higher emissions have been noticed in particleboards bonded with tannin-based adhesives vs. urea-formaldehyde adhesive [17]. Thus far, only two works have been reported about VOC profiles from SE materials of softwood bark pellets, revealing the potentially problematic emissions [18,19].

Based on the highlighted problems’ topicality, the presented study analyzes emissions of VOCs from the binder-less boards obtained from different SE raw lignocellulosics including hemp shives, wheat straw, grey alder wood, and birch wood, and bio-based suberinic acids-bonded particleboard. For VOCs sampling, the headspace solid-phase microextraction (HS-SPME) technique was utilized followed by gas chromatography–mass spectrometry (GC–MS) analyses during 28 days with an interval of 2 weeks.

## 2. Materials and Methods

### 2.1. Raw Lignocellulosics

All lignocellulosics used in the study were grown and obtained in Latvia. Wood species of grey alder (*Alnus incana* (L.) Moench) and birch (*Betula pendula* Roth.) including bark were obtained from “Skrunda” forestry and plywood manufacturer JSC “Latvijas Finieris”, respectively. Wood raw materials without bark were crushed in a knife mill (Retsch SM100, Haan, Germany) to pass a sieve with openings of Ø 2 mm. Agricultural crops of industrial hemp (*Cannabis sativa* L., variety Uso31) and wheat straw (*Triticum aestivum*) were delivered from SIA “NDRA” (Jelgava, Latvia) and ZS “TŪJASMUIŽA” (Salacgrivas County, Latvia), respectively. Agricultural raw materials (only the woody core—shives of the hemp crop—was used in the study) were crushed in a knife mill (CM4000, LAARMANN, Roermond, The Netherlands) to pass a sieve with openings of Ø 10 mm, removing the fraction of ≤0.5 mm.

### 2.2. SE Pretreatment

The crushed raw materials with a moisture content (MC) of 10 ± 2% were pretreated separately in a steam explosion (SE) device of original construction with a 0.5 L batch reactor at the optimal conditions found in the previous studies [20,21]. The SE temperature and time for wood raw materials were 235 °C/1 min, but for agricultural crops, 220 °C/2 min. The pretreated lignocellulosics were collected as received, dried to MC of <12%, and homogenized by crushing in a knife mill (Retsch SM100, Haan, Germany) to pass a 4 mm sieve.

### 2.3. Preparation of Board Samples

#### 2.3.1. Binder-Less Board Fabrication

One-layer binder-less boards were fabricated only from SE lignocellulosics of grey alder (GA), birch (B), hemp shives (HS), and wheat straw (WS) with different densities (700–1300 kg m^−3^) and thicknesses (6 and 12 mm) under the hot-pressing conditions described previously [20,22,23] and shown in Table 1. The binder-less board samples produced under the conditions summarized in Table 1 were selected for this study, because of their best physical-mechanical properties. Some samples of HS and WS boards with a density of 800 kg m^−3^ and thickness of 6 mm were post-treated using overlaying with melamine-impregnated paper under a pressure of 1.4 MPa at 145 °C for 7 min.

#### 2.3.2. Fabrication of Suberinic Acids-Bonded Particleboard (SA-PB)

Bio-based adhesive containing suberinic acids (SA) was obtained from birch wood outer bark, first providing ethanol extraction and then hydrolytic depolymerization in a 3% KOH water solution at 85 °C for 1 h [13]. The obtained wet SA-based adhesive with MC of ~80% (pH 2, solubility in dimethyl sulfoxide 73.0%, acid number 69.5 mg KOH/g) was mixed with fractionated (0.4–2.0 mm) birch wood particles (MC~7%) at a proportion of 21:79 by a mixer (CAT R100C, Paso Robles, CA, USA) and oven-dried at 70 °C to MC ≤ 1%. The prepared dry mixture was hot-pressed by a single-stage press JOOS (Type LAP 40, Gottfried Joos Maschinenfabrik GmbH & Co. KG, Ulm, Germany) at 226 °C for 9.5 min to one-layer board with dimensions and a density of 310 × 310 × 15 mm^3^ and 800 kg m^−3^, respectively.

#### 2.3.3. Control Board Sample

A commercial three-layer particleboard bonded by a typical melamine-urea-formaldehyde adhesive with a thickness of 12 mm and a density of 650 kg m^−3^ was selected for the control board sample and delivered from SIA KRONOSPAN Riga, Latvia.

### 2.4. Determination of VOCs by SPME-GC-TOF-MS Analysis

**Figure 1:**
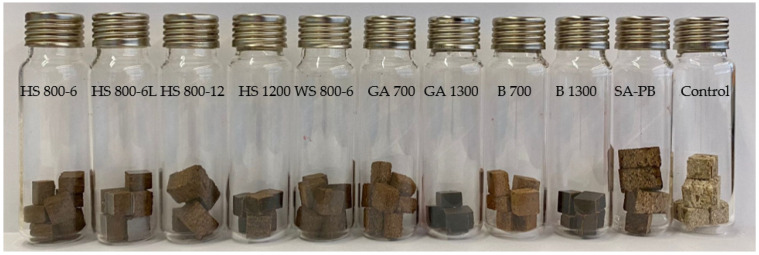
Weighed board specimens in closed vials.

HS-SPME was performed to analyze and compare volatile organic compounds (VOCs) emitted from different board samples, summarized in Table 1. Prior to analysis, the conditioned (RH 65 ± 5%, 20 ± 2 °C) board samples were cut to the rectangular specimens with an approximate area of 7 × 7 mm^2^ from different board sample parts to represent all the board sample. The original thickness of the board specimens was maintained to clarify the impact of board (1) thickness, (2) density, and (3) lamination. The cut board specimens were placed separately in desiccators (Figure 1) and weighed at 2.1 ± 0.05 g for HS-SPME performance after days 1, 14, and 28. Triplicate desiccators with precisely weighed specimens were prepared per board sample.

All samples were analyzed on a gas chromatography time-of-flight mass spectrometry (GC-TOF-MS) LECO Pegasus 4D system, consisting of an Agilent 7890A GC system and a Gerstel multipurpose sampler MPS (Gerstel GmbH, Mulheim an der Ruhr, Germany) coupled with a high-speed TOF-MS detector (LECO, St. Joseph, MI, USA). Sample separation was achieved in a BPX-5 column (30 m, 0.25 mm i.d., 0.25 μm film thickness) (SGE Analytical Science, Melbourn, Australia). The following conditions were applied: the oven temperature started at 30 °C (hold 5 min) and was ramped to 150 °C at a 7 °C/min rate until 300 °C at 20 °C/min; the transfer line and ion source temperature was set to 250 °C; the TOF-MS acquisition rate was 10 spectra/s; the mass range was 30–550 *m*/*z*. Data were collected by ChromaTOF software v.4.22 (LECO) after a solvent peak delay of 30 s.

SPME was performed with a DVB/CAR/PDMS fiber at an equilibration temperature of 40 °C. Samples were equilibrated for 30 min, and then the fiber was exposed for extraction for an additional 30 min. Samples were introduced in the splitless mode in a PTV injector operated at 150 °C and then ramped to 250 °C at 12 °C/s. Helium was used as a carrier gas at a constant flow of 1 mL/min and desorption time of 300 s.

Volatile compounds were identified by comparing their mass spectra with the Adams, NIST, MainLib, and Replib mass spectral libraries. Retention indexes (RI_exp_) were calculated using the retention times of C7–C30 n-alkanes series and further compared with previously published data in the literature (RI_lit_), when available. The results were expressed as GC peak area arbitrary units ×10^7^ (further abbreviated as AU) and percentage (%) of the total GC peak area.

## 3. Results and Discussion

### 3.1. Qualitative Composition of the VOCs from the Obtained Bio-Based Boards

A significant number of different VOCs was detected in the tested bio-based boards by HS-SPME-GC-MS analysis. Table 2, Table 3, Table 4 and Table 5 summarize the main identified chemical compounds. To simplify the tables, only compounds with >0.5% peak area are shown. Differences between RI values obtained experimentally and found in the literature can be explained by small differences between the used chromatographic parameters and differences in the used column stationary phases.

**Figure 2:**
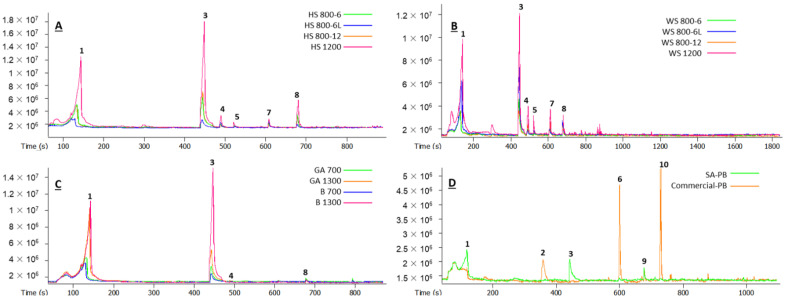
The GC–MS chromatograms (TIC—total ion current) of the VOCs from different board samples: 1—acetic acid; 2—hexanal; 3—furfural; 4—2-furanmethanol; 5—1,3-propanediol; 6—α-pinene; 7—γ-butyrolactone; 8—5-methylfurfural; 9—sylvestrene; 10—nonanal.

The GC–MS chromatograms (TIC) of the VOCs are shown in Figure 2.

The qualitative composition of the VOCs emitted from the binder-less boards fabricated from agricultural biomass was similar. The six chemicals identified in Table 2 and Table 3 covered approximately 95% of the total peak areas in the GC–MS chromatograms. This clearly shows that the main VOCs from HS- and WS-based binder-less boards were the same—acetic acid, furfural, and other furans, as well as some low-molecular-weight aldehydes, alcohols, and organic acids. However, a difference could be observed within the remaining 5% of the peak area. Namely, the number of total detected peaks in the case of HS-based boards (Table 2) was smaller than in the case of WS-based boards (Table 3), regardless of board density or thickness, which means that the VOCs from hemp shives were more uniform. This could be related to the chemical composition of the species, demonstrating a higher amount of extractives and xylan content in raw wheat straw vs. hemp shives [25]. The xylan content remained higher for the wheat straw after SE too. However, the overall hemicelluloses content was very similar (12.54 ± 0.23/13.32 ± 0.22 wt %, respectively, in WS/HS): xylan 11.24/9.53, galactan 0.00/0.00, arabinan 0.58/0.00, mannan 0.00/0.71, acetyl groups 0.72/3.08, and organic acids such as formic acid 2.16/0.47 and levulinic acid 0.93/0.82.

For both HS- and WS-based boards, many other furan derivatives (furoic acid, furfuryl acetate, and methyl furfural isomers), aliphatic carbonyls and carboxyls (levulinic acid), aromatic compounds (eugenol, guaiacol, benzyl alcohol, etc.), and terpenes (linalool) were detected at trace levels. It is worth noting that the highest peak numbers of VOCs were identified in the boards with high-density, particularly in WS-based board, indicating the impact of density on the increased formation of minor compounds. It has been reported that raw hemp shives as the main VOCs emitted α-pinene, limonene, β-pinene, β-myrcene, benzaldehyde, hexanal, and camphene, with generally decreasing emissions over time except for pentanal and hexanal, which increased by at least twice after 14 days [11]. This means that SE processing significantly changes the VOCs profile of lignocellulosics contributing to the drastic formation of furfural and acetic acid because of the degradation of all constituents during the process [26]. There have been reports about the release of acetic acid during the SE process resulting in the furfural formation from degraded C5 sugars, mainly xylose and the formation of 5-HMF from cellulose degradation [27]. Now, it is clear that these substances remain for a long time even in the final composite and proceed to emit, raising concern about the use of the materials.

To reduce the emissions from particleboards, different coatings and overlaying are used, including melamine-impregnated paper used in this study. However, the effect of overlaying on VOCs emissions from agricultural biomass-based boards was not definite. In the case of overlayed HS-based boards, a lower peak area of the main VOCs was observed with a significantly increased peak number (comparison of HS 800-6 and HS 800-6L in Table 2). This means that the overlaying caused new VOCs generated by additional thermal treatment under pressure. However, comparing the overlayed HS-board sample with the non-overlayed HS-board sample of high density, the latter demonstrated an even higher peak number of the VOCs with low concentrations (Table 2 and Figure 2A). Furthermore, a drastic change in the relative area % was observed for the five main compounds: acetic acid, furfuryl acetate, and butyrolactone increased by 2, 5, and 3 times, respectively, but furfural and 5-methyl-furfural decreased by 2 and 4 times, respectively. In the case of overlayed WS-based boards, the main VOCs remained almost in the same proportions with decreased peak number (comparison of WS 800-6 and WS 800-6L in Table 3). The increased individual VOCs emission after the overlaying could be attributed to the applied additional temperature (145 °C) during the overlaying and the chemical composition. For example, the higher contents of xylan and formic acid in the WS sample after SE could result in a higher concentration of furfural during the overlaying process. To some extent, a similar tendency was reported by adding scavengers to the particleboards from different raw materials (pine and poplar), concluding the highest VOC emissions (particularly acids) for pine-based boards, indicating the differences in raw materials producing different VOCs [28].

The woody biomass binder-less board VOCs had fewer dominant compounds—acetic acid and furfural were the dominant ones (Table 4 and Figure 2C), as with the previously described samples. Similarly, γ-butyrolactone and 5-methyl furfural were typical for all binder-less boards, but benzyl alcohol was more characteristic for the woody biomass boards. Table 4 shows that the identified compounds covered approximately 85% of the total peak area in GC–MS chromatograms, despite the fact that the overall number of peaks in the case of grey alder and birch wood materials was smaller than in the case of hemp and wheat. This is primarily due to the comparatively low VOC concentration emitted from the wood-based boards, which hinders identification. However, the lower peak number in wood-based boards was possibly affected by different fabrication conditions including SE and hot-pressing (Table 1). A higher peak number was detected for GA-based boards vs. B-based boards independent of the density, indicating the previously mentioned differences between raw materials. Unlike agricultural crops-based boards, a higher content of minor VOCs was detected in the medium-density boards than in the high-density boards.

Just to compare to available literature data about VOCs from SE materials, 134 substances were found in the SE-treated softwood bark pellets, 53 of which were identified and the main ones being reported as furans (furfural), terpenes (α-pinene), aldehydes (hexanal), and an unknown representing 1–4 × 10^5^ AU [19]. The mentioned study also reported the different VOC levels affected by SE treatment severity when milder conditions (180 °C /5 min) resulted in higher levels of terpenes and aldehydes, whereas the most severely treated (200 °C /5 min) material emitted higher levels of furans.

The commercially produced particleboard sample had a significantly different qualitative profile of the VOCs with terpenes (pinene, sylvestrene, and verbenone), hexanal, and the pleasantly odorous aldehyde nonanal, as the main chemical constituents (Table 5 and Figure 2D), which generally arise from the resins of the softwood particles used for the board production [2,11]. The suberinic acid-bonded particleboard was similar to the previously described binder-less boards, with acetic acid and furans as the main VOCs, arising from the hemicelluloses of the biomass, but also with some compounds present, which were more typical for the commercial sample–hexanal and terpenes. 

It has been proven that the furfural level in SA-PB is highly dependent on the binder preparation parameter; namely, the pH 3 of the binder (pH 2 was for the binder used to produce SA-PB tested in this study) resulted in the highest furfural level [29]. Despite the variety of chemicals emitted from the SA-PB sample, the overall VOC concentration was significantly lower than the binder-less boards, which is explained by the untreated wood particles and the environmentally friendly binder.

**Figure 3:**
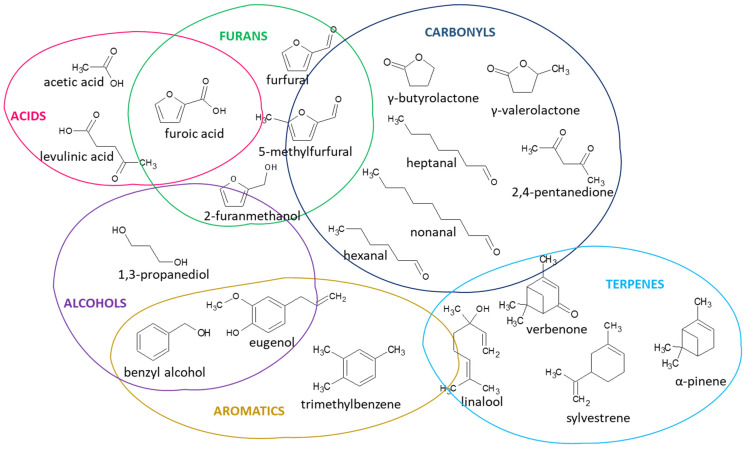
Examples of different chemical species detected in VOCs emitted from the bio-based particleboards.

Figure 3 illustrates the primary chemical compound structures detected in the board samples—organic acids; aliphatic and cyclic carbonyls; furans, which arise from the decomposition of hemicelluloses; aromatic compounds and terpenes, which are more typical extractives. Acetic acid and aldehydes are considered the most odorous and most likely to be perceived by people through olfaction, causing sensory irritation [30]. Terpenes and aldehydes are known to be irritants and pose various health risks [2].

### 3.2. Quantitative VOC Differences between the Bio-Based Boards

**Figure 4:**
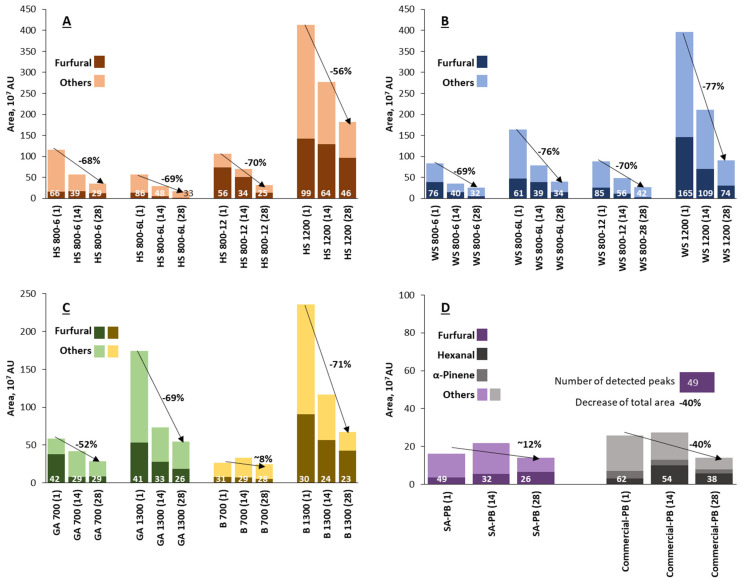
Semi-quantitative comparison of the total VOCs and the furfural fraction emitted from the bio-based boards after days 1, 14, and 28, depending on the raw material, density, thickness, and overlaying. Binder-less boards from (**A**) hemp shives, (**B**) wheat straw, (**C**) grey alder and birch wood, (**D**) suberinic acids-bonded and commercial particleboards.

The total peak areas obtained in the SPME-GC-MS tests were used to semi-quantitatively compare the influence of the boards’ feedstock, density, thickness, and lamination on the VOC emissions during 4 weeks. Figure 4 shows the total VOCs content, expressed as the area units from the chromatograms and the furfural fraction, as it was the most abundant individual compound determined for all binder-less boards, except the Commercial-PB with hexanal and α-pinene as the main VOCs.

The higher density (1200 or 1300 kg m^−3^) of the binder-less boards gave rise to a characteristically higher VOC level. The total VOCs from hemp and wheat binder-less boards had comparable (within 5% difference) content, even though the number of components in the WS 1200 sample was significantly higher than in HS 1200–165 and 99, respectively (see numbers at the base of the bars in Figure 4A,B). There was a strong correlation between the density and the VOC emissions for all raw materials. Namely, the decrease in the density from 1300 to 700 kg m^−3^ meant a decrease in VOCs more than two times. It could be explained that during the pressing at higher densities, VOCs could not escape from the PB matrix and, afterward, slowly evaporated from the sample to a greater extent and for a more extended period. For example, pellets from SE softwood with a density of 750 kg m^−3^ exhibited a lower VOCs level compared to SE biomass immediately after the treatment [19]. The lower-density board samples contain a higher volume and number of pores through which the transportation of VOCs during manufacture is easier than from high-density samples that are highly compressed and, therefore, have few pores. However, as the high-density samples are highly compressed, they also contain a higher number of particles. In this context, in spite of the equal mass of the tested specimens, the lower-density samples contain less material than high-density samples, and, therefore, it also could be attributed to the explanation of the VOCs level that was also approved by another study [31].

**Figure 5:**
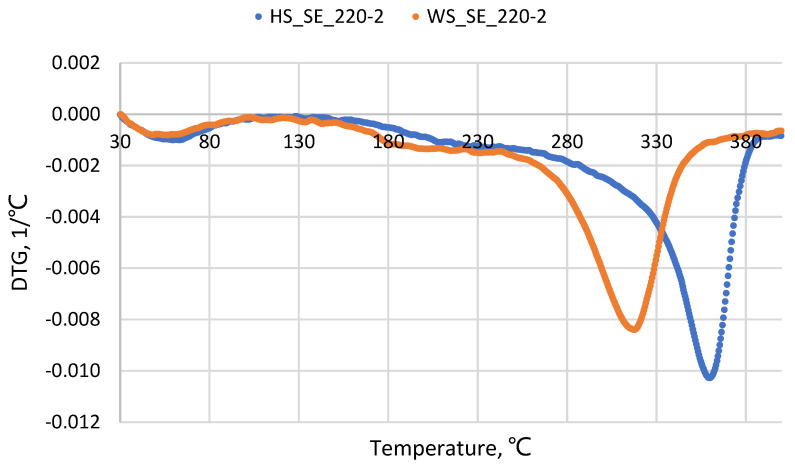
Derived thermogravimetry of steam-exploded (220 °C/2 min) HS and WS samples.

The thicker binder-less boards (12 mm) had similar (within 10% difference) VOC emissions as their 6 mm thick counterparts. Furthermore, slightly higher emissions were released from HS-board samples than from WS-board samples with a significantly higher furfural concentration in 12 mm thick HS-based boards (Figure 4A). Regarding the overlaying effect by melamine-impregnated paper, the goal was achieved for the HS-based board by twice-reducing VOCs; however, the overlayed WS-based board-emitted VOCs increased by a factor of two. This could be explained by the different thermal effect on different lignocellulosics during the overlaying that obviously cause additional VOCs formation especially at elevated temperatures [2]. It was reported that the softening of HS (Uso 31) and WS pretreated by SE at 220 °C for 2 min occurs in the temperature ranges of 163–192 °C and 135–137°C, respectively [25]. Thus, the HS pretreated material is more thermally resistant than WS, which was also confirmed by the derived thermogravimetry analysis shown in Figure 5. This allows the conclusion to be made that the overlaying by melamine-impregnated paper at 145 °C resulted in increased VOCs emitted through the side surfaces only for WS-based board, as well as changed the determined VOCs proportion for both HS- and WS-based boards, as mentioned in a previous subsection. In addition, both total and individual VOCs rate changes depending on varying hot-pressing temperature [32] and indoor air temperature [33] were reported and comply with the results of our study.

It is clear that different kinds of overlaying or coating should be applied to find out the best reducing effect of total VOCs, particularly furfural. For example, overlaying by a phenolic paper of MDF reduced formaldehyde and total VOC emissions by 99% and 88%, respectively, while epoxy powder coatings of the same fiberboard resulted in a similar effect on the reduction of formaldehyde and even better reduction (94%) of VOCs [34]. Sodium metabisulphite was demonstrated as an excellent aldehyde scavenger capable of substantially reducing total VOCs in particleboards with different lignocellulosics [28]. Acetic acid was substantially reduced in parquet products by a solvent-based coating system (PU-1) [5].

The VOCs from the GA- and B-based wood boards had a smaller number of individual components, and the total peak areas of VOCs were lower (Figure 4C), than in the case of hemp and wheat. On the one hand, the lower total VOCs from woody materials could be associated with their earlier production time. However, on the other hand, the VOC emissions were more caused by the freshly cut surfaces before HS-SPME. It was reported that the amount of VOCs highly depends on the manufacturing process of wood-based composite. For example, a lower SE treatment severity of softwood bark resulted in higher VOC levels from produced pellets [19]. It was also reported that total VOCs from MDF were highly influenced by pressing temperature, followed by pressing time [35].

Figure 4 shows that the VOC area units from all four biomass species were almost the same after the 28 days: 36 for HS 800-6, 26 for WS 800-6, 28 for GA 700, and 25 for B 700, indicating a certain equilibrium of persistent VOCs emitted from binder-less boards, unlike the adhesive-bonded ones (SA-PB and commercial-PB). As a comparison, the total VOCs from the commercial-PB were up to 26 AU, which decreased to ~15 AU after 28 days and equaled the SA-PB total VOCs concentration (Figure 4D).

Regarding VOC emission changes over time, the overall trend was a significant decrease in the total VOCs by 52–77% in practically all binder-less board samples after 28 days. Likewise, the number of detected peaks decreased by 23–58%, as the concentration of the minor components dropped below the detection limit. This shows that passive airing during storage removed a large part of the VOCs, which arose from the freshly cut surfaces of the material. A similar tendency was also approved for the pellets from SE softwood bark in enclosed storage, demonstrating a substantial decrease in VOCs after 6 days [18].

The suberinic acid-bonded particleboard did not show a significant decrease in VOCs over time, which were initially low. Namely, the VOCs of the SA-PB sample were comparable to the commercial sample (Figure 4D). Finally, it is worth noting that after 28 days, the overlayed HS-based board released an amount of VOCs comparable to SA-PB and commercial samples with almost no furfural, which is the best result among all binder-less boards investigated during this study.

## 4. Conclusions

It has been shown that the main VOCs from binder-less particleboards made of SE lignocellulosics are acetic acid and furfural, regardless of the raw material species used (hemp shives, wheat straw, grey alder wood, or birch wood). Acetic acid and furfural accounted for up to 80% of the total peak areas of the detected VOCs from the binder-less particleboards. The predominance of acetic acid and furfural was less in the case of the suberinic acid-bonded particleboard. Furthermore, the suberinic acid-bonded particleboard exhibited up to five times lower total VOC emissions than the binder-less boards with a similar density and thickness and was comparable with the commercially produced particleboard. The binder-less particleboards had a strong tendency for decreased (52–77%) VOCs with time, suggesting that simple airing can significantly reduce the harmful VOCs from steam-exploded biomass materials. The lowest VOC emissions were achieved for melamine-impregnated paper-overlayed medium-density boards from SE hemp shives, demonstrating comparable amounts of released total VOCs with the furfural level reduced to zero. The achieved results of the released VOC levels suggest continuing investigation of binder-less boards by seeking an appropriate overlaying or coating to reduce total and specific VOCs.

## Figures and Tables

**Table 1:** Designation and fabrication conditions of board samples provided for VOCs analysis.

Sample	Lignocellulosic	Density, kg m^−3^	Thickness, mm	Pressing T, °C	Pressing τ, min	Overlaying
HS 800-6	Hemp shives	800	6	220	10	–
HS 800-6L	Hemp shives	800	6	220	10	+
HS 800-12	Hemp shives	800	12	210	15	–
HS 1200	Hemp shives	1200	6	175	16	–
WS 800-6	Wheat straw	800	6	220	15	–
WS 800-6L	Wheat straw	800	6	220	15	+
WS 800-12	Wheat straw	800	12	205	18	–
WS 1200	Wheat straw	1200	6	160	12	–
GA 700	Grey alder	700	6	180	16	–
GA 1300	Grey alder	1300	6	150	16	–
B 700	Birch	700	6	180	16	–
B 1300	Birch	1300	6	160	16	–
SA-PB	Birch	800	15	226	9.5	–
Commercial PB	Softwood	650	12	235–220	1	–

**Table 2:** The most abundant identified VOCs and their relative area % from the total peaks in SPME-GC-MS chromatograms of the hemp shives binder-less boards.

Compound	RI_lit_ [24]	RI_exp_	HS 800-6	HS 800-6L	HS 800-12	HS 1200
Acetic acid	580–660	666	16.7 ± 1.3	34.1 ± 5.7	10.5 ± 4.2	28.2 ± 4.1
Furfural	782–847	854	54.8 ± 5.1	24.6 ± 4.1	68.4 ± 6.3	56.0 ± 1.2
2-furanmethanol	863-880	874	2.5 ± 0.5	11.7 ± 3.7	1.8 ± 0.5	2.5 ± 0.8
1,3-propanediol	793–820	890	0.7 ± 0.1	0.6 ± 0.1	<0.5	1.0 ± 0.3
γ-butyrolactone	908–938	940	3.8 ± 0.1	11.8 ± 3.3	3.8 ± 1.5	1.7 ± 1.0
5-methylfurfural	976–990	979	15.6 ± 1.4	4.0 ± 0.8	9.9 ± 3.2	4.7 ± 0.1
Summed area (peak number)	–	–	94.1 (6/66)	86.8 (6/86)	94.4 (5/56)	94.1 (6/99)

**Table 3:** The most abundant identified VOCs and their relative area % from the total peaks in SPME-GC-MS chromatograms of the wheat straw binder-less boards.

Compound	RI_lit_ [24]	RI_exp_	WS 800-6	WS 800-6L	WS 800-12	WS 1200
Acetic acid	580–660	666	30.0 ± 3.8	30.6 ± 4.1	27.1 ± 4.4	24.3 ± 1.7
Furfural	782–847	854	47.0 ± 7.9	54.8 ± 6.2	28.8 ± 4.4	45.2 ± 4.9
2-furanmethanol	863-880	874	4.0 ± 0.4	1.3 ± 0.6	9.4 ± 3.5	6.8 ± 3.0
1,3-propanediol	793–820	890	1.2 ± 0.5	0.7 ± 0.1	1.7 ± 0.5	2.7 ± 1.1
γ-butyrolactone	908–938	940	5.3 ± 3.0	2.4 ± 0.6	10.0 ± 3.4	2.9 ± 0.4
5-methylfurfural	976–990	979	9.9 ± 1.5	5.9 ± 2.0	8.6 ± 2.0	3.3 ± 0.2
Summed area (peak number)	–	–	97.4 (6/76)	95.7 (6/61)	85.6 (6/85)	85.2 (6/165)

**Table 4:** The most abundant identified VOCs and their relative area % from the total peaks in SPME-GC-MS chromatograms of the woody biomass binder-less boards.

Compound	RI_lit_ [24]	RI_exp_	GA 700	GA 1300	B 700	B 1300
Acetic acid	580–660	666	47.3 ± 6.1	61.9 ± 4.6	45.9 ± 9.4	42.3 ± 6.2
Furfural	782–847	854	31.6 ± 10.0	29.9 ± 5.2	31.8 ± 8.2	51.3 ± 6.6
γ-butyrolactone	861–914	940	<0.5	0.6 ± 0.3	1.0 ± 0.4	<0.5
5-methylfurfural	976–990	979	3.6 ± 0.5	1.6 ± 0.3	4.2 ± 0.3	0.7 ± 0.2
Summed area (peak number)	–	–	82.5 (3/42)	93.6 (4/41)	82.9 (4/31)	94.0 (3/30)

**Table 5:** The most abundant identified VOCs and their relative area % from the total peaks in SPME-GC-MS chromatograms of the adhesive-bonded particleboards.

Compound	RI_lit_ [24]	RI_exp_	SA-PB	Commercial PB
Acetic acid	580–660	666	14.8 ± 10.1	5.2 ± 1.6
Hexanal	762–822	813	<0.5	13.2 ± 5.7
Furfural	782–847	854	21.8 ± 11.0	<0.5
Heptanal	856–909	913	n ^1^	0.9 ± 0.4
α-pinene	923–934	934	0.9 ± 0.2	38.7 ± 16.2
5-methylfurfural	976–990	979	13.1 ± 1.9	n
1,2,4-trimethylbenzene	956–1013	999	1.9 ± 0.3	n
Sylvestrene	1016–1018	1033	<0.5	1.5 ± 0.3
Nonanal	1069–1130	1114	0.9 ± 0.4	2.4 ± 0.7
Verbenone	1164–1217	1224	n	0.8 ± 0.2
Summed area (peak number)	–	–	53.4 (6/49)	62.7 (7/62)

^1^ not detected.

## Data Availability

Not applicable.
